# α,β,γ− Neutrosophic aggregation operators and their applications in the software site selection

**DOI:** 10.1016/j.heliyon.2024.e31417

**Published:** 2024-05-20

**Authors:** Sumbal Ali, Muhammad Rahim, Sanaa A. Bajri, Sadique Ahmad, Rabab Alharbi, Hamiden Abd El-Wahed Khalifa

**Affiliations:** a Department of Mathematics and Statistics, Hazara University, Mansehra, 21300, KPK, Pakistan; b Department of Mathematical Sciences, College of Science, Princess Nourah Bint Abdulrahman University, P.O. Box 84428, Riyadh, 11671, Saudi Arabia; c EIAS: Data Science and Blockchain Laboratory, College of Computer and Information Sciences, Prince Sultan University, Riyadh, 11586, Saudi Arabia; d Department of Mathematics, College of Science, Qassim University, Buraydah, 51452, Saudi Arabia; e Department of Operations and Management Research, Faculty of Graduate Studies of Statistical Research, Cairo University, Giza, 12613, Egypt

**Keywords:** α,β,γ-neutrosophic sets, Operational laws, Aggregation operators, Decision making, Optimization

## Abstract

In this paper, we expended the concept of neutrosophic sets (NS) by introducing the idea α,β,γ− neutrosophic set (α,β,γ− NS). The existing models under conventional NSs, fail to adequately address the management of membership degree influence during the aggregation process. While the proposed framework manages the influence of membership degree (MD), indeterminacy membership degree (IMD), and non-membership degree (NMD) by incorporating parameters α, β, and γ. Furthermore, we defined some fundamental operational laws for α,β,γ− NSs and introduced a series of aggregation operators (AOs) to effectively combine α,β,γ− neutrosophic information. Based on these AOs, a new Multiple Criteria Decision Making (MCDM) model is proposed for solving real-life decision-making (DM) challenges.

An illustrative case study is presented to showcase the effectiveness of the proposed model in selecting an optimal location for a software office. The article concludes by validating the proposed model's authenticity and effectiveness through a comparative analysis with existing approaches.

## 1 Introduction

Multi-Criteria Decision Making (MCDM) is a decision-making paradigm that involves evaluating and selecting alternatives based on multiple conflicting criteria or objectives. Unlike traditional decision-making methods that consider only one criterion, MCDM acknowledges the complex nature of real-world decision problems where decisions often need to balance multiple, sometimes conflicting, objectives. In MCDM, decision-makers aim to identify the best possible alternative(s) by systematically assessing the performance of each alternative across multiple criteria. Various techniques are employed in MCDM to facilitate this evaluation process, including weighted sum methods, analytic hierarchy process (AHP) [1], technique for order preference by similarity to ideal solution (TOPSIS) [2], and outranking methods like PROMETHEE [3]. MCDM finds applications in diverse fields such as engineering [4], management [5], finance [6], environmental planning [7], and healthcare [8], where decisions involve considerations of cost, risk, time, quality, and other factors. By providing a systematic and structured approach to decision-making, MCDM helps decision-makers navigate complex decision scenarios, prioritize objectives, and identify robust solutions that balance conflicting criteria effectively. Moreover, MCDM offers decision support tools and methodologies to address uncertainty, facilitate consensus-building among stakeholders, and enhance the transparency and accountability of decision-making processes. Zadeh [9] initiated a powerful mathematical framework called fuzzy set FS, characterized by degree of membership for solving the uncertainty and vagueness in decision making. In control system, pattern recognition, artificial intelligence, decision making, linguistics and more field fuzzy sets are use. Zadeh [10,11] presented the idea interval-valued FS (IVFS). In FS we only deal the degree of acceptant which is also called membership degree, since there is no information regarding to rejection, therefore, Atanassov [12] combined non-membership function with membership function and introduced intuitionistic fuzzy set (IFS), by constraint that sum DM and NMD less than or equal to 1. Under the environment of IFS, some AOs were developed by Zhao et al. [13] and Tan et al. [14,15]. In some real-life scenarios, the total sum of the MD and NMD may be exceeds 1. In such situations IFSs are unable to deal the scenarios. To address these limitations, Yager [16] introduced Pythagorean FSs (PFSs) with the conditions that the square sum of MD and NMD less than or equal to 1. The concept of IFS and PFS was further generalized by introducing q-rung orthopair fuzzy sets (*q-*ROFSs) [17] such that ${MD}^{q}+{NMD}^{q}\leq 1$. Sheikh and Mandal [18] extended the idea of *q-*ROFSs by presenting the idea of p,q− quasirung orthopair fuzzy sets (p,q− QOFSs) such that ${MD}^{p}+{NMD}^{q}\leq 1$. The preceding dialogue highlights that existing approaches are built upon considering Membership Degree (MD) and Non-Membership Degree (NMD), neglecting the aspect of indeterminacy Membership Degree (IMD) in the evaluation of an object. In response to this oversight, Smarandache [19] addressed the gap by introducing neutrosophic sets, encompassing MD, IMD, and NMD, with the condition that the sum of MD, IMD, and NMD is constrained to be less than or equal to 3.

### 1.1 Literature review

NSs present distinct advantages by incorporating IMD, thus offering a more comprehensive representation of uncertainty compared to traditional fuzzy sets. The inclusion of IMD enhances the framework's capability to model and handle ambiguity, accommodating situations where the precise membership status of an element is indeterminate. With flexibility in representation through three parameters MD, IMD, and NMD sets provide a nuanced and adaptable approach to expressing complex information, particularly beneficial in scenarios where conventional fuzzy sets may fall short. Their versatility extends to handling incomplete or partial information and seamlessly integrating with other set theories, fostering interoperability. The expressiveness of neutrosophic sets makes them applicable in diverse fields, including artificial intelligence, data mining, and decision support systems, addressing the complexities of real-world problems with inherent uncertainty. A number of scholars have proposed different methods for using Neutrosophic Sets (NSs). For examples, Garg and Nancy [20] developed a series hybrid aggregation operators, including arithmetic and geometric operators for single and interval-valued neutrosophic numbers, with an investigation into their desirable properties such as idempotency, boundedness, and monotonicity. Sodenkamp et al. [21] proposed a method to represent multi-source uncertainty in estimates from diverse domain experts in MCDM problems, along with a methodology for integrating these measures into a unified decision support procedure. Liu and Tang [22] synergized power and Geometric Weighted Average operators into interval-valued neutrosophic sets (IVNS) and introduced various aggregation operators to aggregated interval neutrosophic information. Garg and Nancy [23] presented operators designed for the aggregation of single-valued neutrosophic information and applied these operators to address MCDM problems. Senapati [24] suggested Aczel-Alsina operations and AOs tailored for single-valued Neutrosophic Sets (SVNSs), integrating conventional Aczel-Alsina methods to aggregate neutrosophic information. For additional insights into aggregation operators, distance measures, and distance-based approaches, readers are encouraged to refer to the works cited in Refs. [[25], [26], [27], [28], [29], [30], [31], [32], [33], [34]].

The gap between α,β,γ− neutrosophic sets and neutrosophic sets lies primarily in the level of granularity and control offered in representing uncertainty and imprecision. Neutrosophic sets provide a broad framework encompassing truth-membership, indeterminacy-membership, and falsity-membership degrees, but lack specific parameters for fine-tuning the influence of each degree, limiting the precision in modeling uncertainty. On the other hand, α,β,γ− neutrosophic sets introduce parameters α, β, and γ to govern the influence of membership, non-membership, and indeterminacy degrees, respectively. These parameters offer greater granularity and control, allowing decision-makers to adjust the influence of each degree according to the specific requirements of the problem domain. By adjusting α, β, and γ, decision-makers can tailor the representation of uncertainty with more precision, leading to more accurate and nuanced decision-making outcomes compared to traditional neutrosophic sets. Thus, while neutrosophic sets provide a general framework for handling uncertainty, α,β,γ− neutrosophic sets offer a more advanced and customizable approach, bridging the gap between theoretical models and practical applications in decision-making and artificial intelligence.

### 1.2 Motivations

The utilization of existing neutrosophic sets and their aggregation operators has garnered considerable interest across various fields, attracting the attention of numerous researchers. Despite their widespread application, these aggregations lack parameters to effectively regulate the influence of membership degrees in the DM process. In addressing this limitation, our paper introduces a novel framework, namely the α,β,γ− neutrosophic set, as an extension of the established neutrosophic set. This innovative structure incorporates three key parameters: α, which governs the impact of membership degree (MD); β, regulating non-membership degree (NMD); and γ, controlling the indeterminacy membership degree (IMD) of neutrosophic information. This enhancement enables a more nuanced and customizable approach to managing the intricacies of membership influence during the DM process, providing a valuable contribution to the field of neutrosophic set theory.

From this vantage point, we offer an example to further elucidate the scenario. Suppose the project manager is assessing the risk of delays due to inclement weather using α,β,γ− neutrosophic sets. The manager assigns α=1 to expert meteorological forecasts, indicating a high level of confidence in their accuracy and thus a higher influence on MD. Meanwhile, the manager assigns higher β=3 and γ=2 values to historical weather data and probabilistic models, indicating a lower reliability and relevance to the project, and thus a higher influence on NMD and IMD, respectively.

By adjusting α, β, and γ, the manager can achieve a more accurate and contextually relevant aggregation of risk information, leading to better-informed decisions about scheduling and resource allocation to mitigate these risks effectively. This allows the manager to consider the varying degrees of uncertainty associated with different sources of information, aligning with the specified influence of α, β, and γ in the aggregation process.

### 1.3 Contributions

1) To define α,β,γ− NSs and their basic operational laws.
2) To develop Averaging and Geometric operators based on α,β,γ− NSs and demonstration of their fundamental properties.
3) To propose a new MCDM model based on the proposed AOs to solve DM problems.

**Fig. 1:**
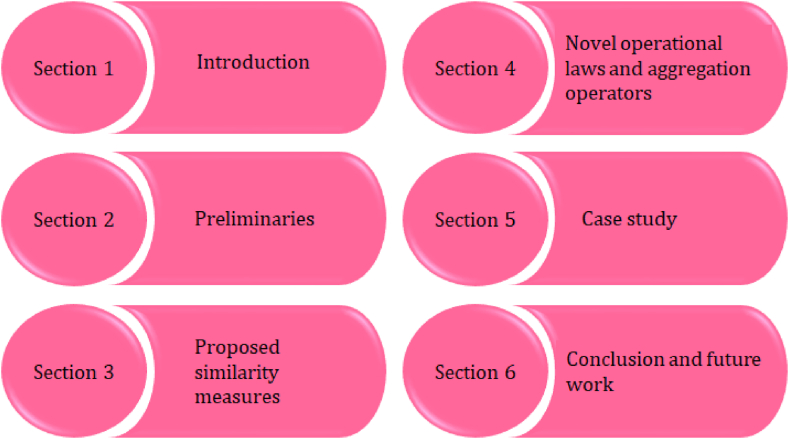
Layout of the proposed work.

This manuscript is structured into six sections to comprehensively address the proposed framework: Section 1 serves as the Introduction, setting the stage for the subsequent discussions. Section 2 delves into preliminaries, establishing the foundational knowledge necessary for understanding the forthcoming concepts. Section 3 elucidates the concept of α,β,γ− neutrosophic sets (α,β,γ− NSs) and details their basic operations. In Section 4, we introduce Aggregation Operators (AOs) grounded in α,β,γ− neutrosophic numbers. The fifth section goes on to showcase a step-wise algorithm for a mathematical model, utilizing these AOs. An illustrative example, centered around the selection of an optimal software office location, is presented to underscore the flexibility, reliability and sensitivity of our proposed model. Finally, Section 6 encapsulates the manuscript with a conclusive summary. The graphical layout is presented in Fig. 1.

Table 1 contains a listing of abbreviations utilized in this article.

**Table 1:** List of abbreviations.

p,q− quasirung orthopair fuzzy set	p,q− QOFS
α,β,γ− neutrosophic sets	α,β,γ− NSs
α,β,γ− neutrosophic numbers	α,β,γ− NNs
α,β,γ− neutrosophic weighted averaging	α,β,γ− NWA
α,β,γ− neutrosophic order weighted averaging	α,β,γ− NOWA
α,β,γ− neutrosophic weighted geometric	α,β,γ− NWG
α,β,γ− neutrosophic order weighted geometric	α,β,γ− NOWG
Membership degree	MD
Neutral membership degree	NED
Non-membership degree	NMD
Multi-criteria decision making	MCDM
Averaging operators	AOs

## 2 Preliminaries

Definition 1 [12] Let M be a fixed set and a mathematical structure of IFS I for an element m∈M is expressed as follows:

(1) $$I=\left\{\left\langle m,\psi_{I}\left(m\right),\theta_{I}\left(m\right)\right\rangle |m\in M\right\}$$

In Equation (1), $\psi_{I}\left(m\right)$ and $\theta_{I}\left(m\right)$ express MD and NMD of an element m∈M, such that $\left(\psi_{I}\left(m\right)\right)+\left(\theta_{I}\left(m\right)\right)≼1$.

Definition 2 [16] Let M be a fixed set. A mathematical structure of a PFS P over an element m∈M is defined as follows:

(2) $$P=\left\{\left\langle m,\psi_{P}\left(m\right),\psi_{P}\left(m\right)\right\rangle :m\in M\right\}$$

In Equation (2), $\psi_{P}\left(m\right)$ and $\theta_{P}\left(m\right)$ represent MD and NMD of an element m∈M such that ${\left(\psi_{P}\left(m\right)\right)}^{2}+{\left(\theta_{P}\left(m\right)\right)}^{2}≼1$.

Definition 3 [17] Let M be a fixed set. A mathematical structure of a q-ROFS over an element m∈M can be defined as follows:

(3) $$Q=\left\{\left\langle m,\psi_{Q}\left(m\right),\theta_{Q}\left(m\right)\right\rangle |m\in M\right\}$$

In Equation (3), $\psi_{Q}\left(m\right)$ and $\theta_{Q}\left(m\right)$ represent MD and NMD of an element m∈M such that ${\left(\psi_{Q}\left(m\right)\right)}^{q}+{\left(\theta_{Q}\left(m\right)\right)}^{q}≼1$ for all q≥1.

Definition 4 [18] Let M be a fixed set. A mathematical structure of a p,q− QOFS for an element m∈M can be represented as:

(4) $$R=\left\{\left\langle m,\psi_{R}\left(m\right),\theta_{R}\left(m\right)\right\rangle |m\in M\right\}$$

In Equation (4), $\psi_{R}\left(m\right)$ and $\theta_{R}\left(m\right)$ denotes the MD and NMD of a component m∈M in R such that ${\left(\psi_{R}\left(m\right)\right)}^{p}+{\left(\theta_{R}\left(m\right)\right)}^{q}≼1$ for all p, q≽1.

Definition 5 [19] Let M be a fixed set. A mathematical structure of a neutrosophic set J is defined as:

(5) $$J=\left\{\left\langle m,\psi_{J}\left(m\right),\eta_{J}\left(m\right),\theta_{J}\left(m\right)\right\rangle |m\in M\right\}$$

In Equation (5) $\psi_{J}\left(m\right)$, $\eta_{J}\left(m\right)$ and $\theta_{J}\left(m\right)$ express truth, indeterminacy, and falsity degree, such that $\left(\psi_{J}\left(m\right)\right)+\left(\eta_{J}\left(m\right)+\left(\theta_{J}\left(m\right)\right)\right)\leq 3$.

Definition 6 [35] The score function of neutrosophic number J can be determined as follows:

(6) $$\text{sc}\left(J\right)=2+\left(\psi_{J}\left(m\right)\right)-\left(\eta_{J}\left(m\right)\right)-\left(\theta_{J}\left(m\right)\right)$$

where 0≤sc(J)≤3.

The accuracy function [36] of neutrosophic number J can be determined as follows:

(7) $$\text{ac}\left(J\right)=\left(\psi_{J}\left(m\right)\right)-\left(\theta_{J}\left(m\right)\right)$$

where 0≤ac(J)≤3. The score and accuracy function defined in Equation (6), (7) can be used to compare neutrosophic numbers.

## 3 α,β,γ− Neutrosophic sets and their operational laws

Within this section, we have introduced several operational laws for α,β,γ− neutrosophic sets.

Definition 7 Let M be a universal set. A α,β,γ− NS an element m∈M can be defined as follows:

(8) $$D=\left\{\left\langle m,\psi_{D}\left(m\right),\eta_{D}\left(m\right),\theta_{D}\left(m\right)\;\right\rangle |m\in M\right\}$$

In Equation (8), $\psi_{D}\left(m\right)$, $\eta_{D}\left(m\right)$ and $\theta_{D}\left(m\right)\in \left[0,1\right]$ represents the truth, indeterminacy and falsity degree of an element m∈M in set D such that ${\left(\psi_{D}\left(m\right)\right)}^{\alpha }+{\left(\eta_{D}\left(m\right)\right)}^{\gamma }+{\left(\theta_{D}\left(m\right)\right)}^{\beta }≼3$.

Remark 1 The parameters α, β and γ are positive integers such that

1. α≺β, α≻β or α=β,
2. γ=LCM(α,β).

For simplicity we can represented as α,β,γ− NN as (ψ,η,θ) such that $\psi^{\alpha }+\eta^{\gamma }+\theta^{\beta }≼3$.

α,β,γ− NS allow individuals and organizations to adjust the parameters α, β, and γ to reflect their values and goals effectively. By setting appropriate values for α, β, and γ, decision-makers can ensure that their decisions align with their ethical principles and long-term objectives. For example, setting higher values of α might prioritize certain moral principles, while adjusting β and γ can account for situational constraints and personal preferences. Also, α,β,γ− NS sets support continuous learning and adaptation by allowing decision-makers to adjust parameters in response to new information or changing circumstances.

Definition 8 The degree of hesitancy of α,β,γ− NN can defined as follows:

(9) $$\Delta \left(m\right)=\sqrt[{\gamma }]{1-{\left(\psi_{D}\left(m\right)\right)}^{\alpha }-{\left(\eta_{D}\left(m\right)\right)}^{\gamma }-{\left(\theta_{D}\left(m\right)\right)}^{\beta }}$$

in Equation (9), α, β are positive integers such that γ=LCM(α,β).

Definition 9 The score function for α,β,γ− NN D=(ψ,η,θ) is defined as follows:

(10) $$\text{sc}\left(D\right)=2+{\left(\psi \right)}^{\alpha }-{\left(\eta \right)}^{\;\gamma }-{\left(\theta \right)}^{\beta }$$

where 0≼sc(D)≼3.

Definition 10 The accuracy function for α,β,γ− NN D=(ψ,η,θ) is defined as follows:

(11) $$\text{ac}\left(D\right)=\psi^{\alpha }+\eta^{\gamma }+\theta^{\beta }$$

where 0≤ac(D)≤3. The score and accuracy functions defined in Equations (10), (11) are more generalized the existing score and accuracy functions.

Definition 11 Let $D_{1}=\left(\psi_{1},\eta_{1},\theta_{1}\right)$ and $D_{2}=\left(\psi_{2},\eta_{2},\theta_{2}\right)$ be two α,β,γ− NNs, then

1. If $\text{sc}\left(D_{1}\right)≺\text{sc}\left(D_{2}\right)$, then $D_{1}≺D_{2}$,
2. If $\text{sc}\left(D_{1}\right)≻\text{sc}\left(D_{2}\right)$, then $D_{1}≻D_{2}$,
3. If $\text{sc}\left(D_{1}\right)=\text{sc}\left(D_{2}\right)$, then
4. If $\text{ac}\left(D_{1}\right)≺\text{ac}\left(D_{2}\right)$, then $D_{1}≺D_{2}$,
5. If $\text{ac}\left(D_{1}\right)≻\text{ac}\left(D_{2}\right)$, then $D_{1}≻D_{2}$,
6. If $\text{ac}\left(D_{1}\right)=\text{ac}\left(D_{2}\right)$, then $D_{1}=D_{2}$.

Definition 12 For any three α,β,γ− NNs D=(ψ,η,θ), $D_{1}=\left(\psi_{1},\eta_{1},\theta_{1}\right)$ and $D_{2}=\left(\psi_{2},\eta_{2},\theta_{2}\right)$ where α, β≽1, γ=LCM(α,β) and ξ≻0. Then their operations are defined as follows:

1. $D_{1}⊕D_{2}=\left(\begin{array}{c} \sqrt[{\alpha }]{{\left(\psi_{1}\right)}^{\alpha }+{\left(\psi_{2}\right)}^{\alpha }-{\left(\psi_{1}\right)}^{\alpha }{\left(\psi_{2}\right)}^{\alpha }},\\ \eta_{1}\eta_{2},\theta_{1}\theta_{2} \end{array}\right)$,
2. $D_{1}⊗D_{2}=\left(\begin{array}{c} \psi_{1}\psi_{2},\sqrt[{\;\gamma }]{{\left(\eta_{1}\right)}^{\gamma }+{\left(\eta_{2}\right)}^{\gamma }-{\left(\eta_{1}\right)}^{\gamma }{\left(\eta_{2}\right)}^{\gamma }},\\ \sqrt[{\beta }]{{\left(\theta_{1}\right)}^{\beta }+{\left(\theta_{2}\right)}^{\beta }-{\left(\theta_{1}\right)}^{\beta }{\left(\theta_{2}\right)}^{\beta }} \end{array}\right)$,
3. $\xi D=\left(\sqrt[{\alpha }]{1-{\left(1-{\left(\psi \right)}^{\alpha }\right)}^{\xi }},{\left(\eta \right)}^{\xi },{\left(\theta \right)}^{\xi }\right)$,
4. $D^{\xi }$ = $\left(\begin{array}{c} {\left(\psi \right)}^{\xi },\sqrt[{\;\gamma }]{1-{\left(1-{\left(\eta \right)}^{\gamma }\right)}^{\xi }},\\ \sqrt[{\beta }]{1-{\left(1-{\left(\theta \right)}^{\beta }\right)}^{\xi }} \end{array}\right)$,
5. $D_{1}\cup D_{2}=\left(\max\left(\psi_{1},\psi_{2}\right),\min\left(\eta_{1},\eta_{2}\right),\min\left(\;\theta_{1},\theta_{2}\right)\right)$,
6. $D_{1}\cap D_{2}\left(\min\left(\psi_{1},\psi_{2}\right),\max\left(\eta_{1},\eta_{2}\right),\max\left(\;\theta_{1},\theta_{2}\right)\right)$,
7. $D^{C}=\left(\theta ,\eta ,\psi \right)$,
8. $D_{1}≼D_{2}$ if and only if $\psi_{1}≼\psi_{2},\theta_{1}≽\theta_{2},\eta_{1}≽\eta_{2}$.

Example 1 Consider two α,β,γ− NNs $D_{1}=\left(0.40,0.30,0.20\right)$ and $D_{2}=\left(0.80,0.10,0.10\right)$ where α=β=5, γ=LCM(5,5)=5 and ξ=0.5, then

$$D_{1}⊕D_{2}=\left(\sqrt[{\alpha }]{{\left(\psi_{1}\right)}^{\alpha }+{\left(\psi_{2}\right)}^{\alpha }-{\left(\psi_{1}\right)}^{\alpha }{\left(\psi_{2}\right)}^{\alpha }},\eta_{1}\eta_{2},\theta_{1}\theta_{2}\right)=\left(\begin{array}{c} \sqrt[{5}]{{\left(0.4\right)}^{5}+{\left(0.8\right)}^{5}-{\left(0.4\right)}^{5}{\left(0.8\right)}^{5}},\\ 0.3\times 0.1,0.2\times 0.1 \end{array}\right)$$

=(0.8033,0.0334,0.0212).

$$D_{1}⊗D_{2}=\left(\begin{array}{c} \psi_{1}\psi_{2},\sqrt[{\gamma }]{{\left(\eta_{1}\right)}^{\gamma }+{\left(\eta_{2}\right)}^{\gamma }-{\left(\eta_{1}\right)}^{\gamma }{\left(\eta_{2}\right)}^{\gamma }},\\ \sqrt[{\beta }]{{\left(\theta_{1}\right)}^{\beta }+{\left(\theta_{2}\right)}^{\beta }-{\left(\theta_{1}\right)}^{\beta }{\left(\theta_{2}\right)}^{\beta }} \end{array}\right)=\left(\begin{array}{c} 0.4\times 0.8,\sqrt[{\;5}]{{\left(0.3\right)}^{5}+{\left(0.1\right)}^{5}-{\left(0.3\right)}^{5}{\left(0.1\right)}^{5}},\\ \sqrt[{\;5}]{{\left(0.2\right)}^{\;5}+{\left(0.1\right)}^{5}-{\left(0.2\right)}^{5}{\left(0.1\right)}^{5}} \end{array}\right)$$

=(0.3200,0.3002,0.2012).

$$\xi D=\left(\sqrt[{\alpha }]{1-{\left(1-{\left(\psi \right)}^{\alpha }\right)}^{\xi }},{\left(\eta \right)}^{\xi },{\left(\theta \right)}^{\xi }\right)=\left(\sqrt[{5}]{1-{\left(1-{\left(0.4\right)}^{5}\right)}^{0.5}},{\left(0.3\right)}^{0.5},{\left(0.2\right)}^{0.5}\right)$$

=(0.3484,0.5477,0.4472).

$$D^{\xi }=\left({\left(\psi \right)}^{\xi },\sqrt[{\;\gamma }]{1-{\left(1-{\left(\eta \right)}^{\gamma }\right)}^{\xi }},\sqrt[{\beta }]{1-{\left(1-{\left(\theta \right)}^{\beta }\right)}^{\xi }}\right)=\left(\begin{array}{c} {\left(0.40\right)}^{0.5},\sqrt[{\;5}]{1-{\left(1-{\left(0.30\right)}^{\;5}\right)}^{0.5}},\\ \sqrt[{5}]{1-{\left(1-{\left(0.20\right)}^{5}\right)}^{0.5}} \end{array}\right)$$

=(0.6324,0.2612,0.1741).

$$D_{1}\cup D_{2}=\left(\max\left(\psi_{1},\psi_{2}\right),\min\left(\eta_{1},\eta_{2}\right),\min\left(\theta_{1},\theta_{2}\right)\right)=\left(\max\left(0.40,0.80\right),\min\left(0.30,0.10\right),\min\left(\;0.20,0.10\right)\right)$$

=(0.80,0.10,0.10).

$$D_{1}\cap D_{2}=\left(\min\left(\psi_{1},\psi_{2}\right),\max\left(\eta_{1},\eta_{2}\right),\max\left(\theta_{1},\theta_{2}\right)\right)=\left(\min\left(0.40,0.80\right),\max\left(0.30,0.10\right),\max\left(0.20,0.10\right)\right)$$

=(0.40,0.30,0.20).

$$D^{C}={\left(\psi ,\eta ,\theta \right)}^{C}={\left(0.40,0.30,0.20\right)}^{C}=\left(0.20,0.30,0.40\right)\text{.}$$

Theorem 1 *For any three*α,β,γ−*NNs*D=(ψ,η,θ), $D_{1}=\left(\psi_{1},\eta_{1},\theta_{1}\right)$*and*$D_{2}=\left(\psi_{2},\eta_{2},\theta_{2}\right)$*and*ξ, $\xi_{1}$, $\xi_{2}≻0$, *then the following properties holds*.

1. $D_{1}⊕D_{2}=D_{2}⊕D_{1}$,
2. $D_{1}⊗D_{2}=D_{2}⊗D_{1}$,
3. $\xi \left(D_{1}⊕D_{2}\right)=\xi D_{1}⊕\xi D_{2}$,
4. $\left(\xi_{1}⊕\xi_{2}\right)D=\xi_{1}D⊕\xi_{2}D$.

**Proof.** Since $D_{1}⊗D_{2}=\left(\begin{array}{c} \psi_{1}\psi_{2},\sqrt[{\;\gamma }]{{\left(\eta_{1}\right)}^{\gamma }+{\left(\eta_{2}\right)}^{\gamma }-{\left(\eta_{1}\right)}^{\gamma }{\left(\eta_{2}\right)}^{\gamma }},\\ \sqrt[{\beta }]{{\left(\theta_{1}\right)}^{\beta }+{\left(\theta_{2}\right)}^{\beta }-{\left(\theta_{1}\right)}^{\beta }{\left(\theta_{2}\right)}^{\beta }} \end{array}\right)$.

$$=\left(\begin{array}{c} \psi_{2}\psi_{1},\sqrt[{\;\gamma }]{{\left(\eta_{2}\right)}^{\gamma }+{\left(\eta_{1}\right)}^{\gamma }-{\left(\eta_{2}\right)}^{\gamma }{\left(\eta_{1}\right)}^{\gamma }},\\ \sqrt[{\beta }]{{\left(\theta_{2}\right)}^{\beta }+{\left(\theta_{1}\right)}^{\beta }-{\left(\theta_{2}\right)}^{\beta }{\left(\theta_{1}\right)}^{\beta }} \end{array}\right)=D_{1}⊗D_{2}\text{.}$$

Similarly, 1, 3, and 4 can be proved accordingly.

## 4 Proposed AOs based on α,β,γ− NSs

In this section, we proposed a series of AOs such as α,β,γ− neutrosophic weighted averaging (α,β,γ− NWA), α,β,γ− neutrosophic ordered weighted averaging (α,β,γ− NOWA), α,β,γ-neutrosophic weighted geometric (α,β,γ− NWG), and α,β,γ− netrosophic ordered weighted geometric operators to aggregated α,β,γ-netrosophic information.

### 4.1 α,β,γ− NWA operator

Definition 13 For any collection of α,β,γ− NNs ${Η}_{i}$ = $\left(\psi_{i},\eta_{i},\theta_{i}\right)$ for variable i ranging from 1 to n, having weight vector $\tau_{i}$ with $\tau_{i}\in \left[0,1\right]\;\text{such}\;\text{that}\;\sum_{i=1}^{n}\tau_{i}=1$. Then mapping α,β,γ− NWA: $\Lambda^{n}⟶\Lambda$, where Λ represent the collection of α,β,γ− NNs, called α,β,γ− NWA operator is given by:

(12) $$\alpha ,\beta ,\gamma -\left(D_{1},D_{2},\ldots .,D_{n}\right)={⊕}_{i=1}^{n}\left(\tau_{i}\;D_{i}\right)$$

Theorem 2 *Suppose that*$D_{i}=\left(\psi_{i},\eta_{i},\theta_{i}\right)$*be the collection of*α,β,γ−*NNs for all*i=1,2,...,n. *The aggregation results of*α,β,γ−*NWA operator is also*α,β,γ−*NNs and can be represented as follows*:

(13) $$\alpha ,\beta ,\gamma -\text{NWA}\left(D_{1},D_{2},\ldots ,D_{n}\right)=\left(\begin{array}{c} \sqrt[{\alpha }]{1-{\Pi_{i=1}^{n}\left(1-\psi_{i}^{\alpha }\right)}^{\tau_{i}}},\\ \Pi_{i=1}^{n}\eta_{i}^{\tau_{i}},\\ \Pi_{i=1}^{n}\theta_{i}^{\tau_{i}} \end{array}\right)$$

where $\tau_{i}$ is the weight vector with the condition $\sum_{i=1}^{n}\tau_{i}=1$ and α is any positive integer.

**Proof.** Mathematical induction is employed to prove the validity of Theorem 2.

**Step 1.** For n=2, $D_{1}=\left(\psi_{1},\eta_{1},\theta_{1}\right)$ and $D_{2}=\left(\psi_{2},\eta_{2},\theta_{2}\right)$.

$$\alpha ,\beta ,\gamma -\;\text{NWA}\;\left(D_{1},D_{2}\right)=\left(\tau_{1}D_{1}⊕\tau_{2}D_{2}\right)=\left(\sqrt[{\alpha }]{1-{\left(1-\psi_{1}^{\alpha }\right)}^{\tau_{1}}},{\left(\eta_{1}\right)}^{\tau_{1}},{\left(\theta_{1}\right)}^{\tau_{1}}\right)$$

$$⊕\left(\sqrt[{\alpha }]{1-{\left(1-\psi_{2}^{\alpha }\right)}^{\tau_{2}}},{\left(\eta_{2}\right)}^{\tau_{2}},{\left(\theta_{2}\right)}^{\tau_{2}}\right)=\left(\begin{array}{c} \sqrt[{\alpha }]{1-{\left(1-\psi_{1}^{\alpha }\right)}^{\tau_{1}}{\left(1-\psi_{2}^{\alpha }\right)}^{\tau_{2}}},{\left(\eta_{1}\right)}^{\tau_{1}}{\left(\eta_{2}\right)}^{\tau_{2}},\\ {\left(\theta_{1}\right)}^{\tau_{1}}{\left(\theta_{2}\right)}^{\tau_{2}} \end{array}\right)$$

$$=\left(\sqrt[{\alpha }]{1-{\Pi_{i=1}^{2}\left(1-\psi_{i}^{\alpha }\right)}^{\tau_{i}}},\Pi_{i=1}^{2}\eta_{i}^{\tau_{i}},\Pi_{i=1}^{2}\theta_{i}^{\tau_{i}}\;\right)\text{.}$$

Thus, the result is valid for n=2.

**Step 2.** We suppose that Equation (13) is valid for n=k i.e.,

(14) $$\alpha ,\beta ,\gamma -\text{NWA}\left(D_{1},D_{2},\ldots ,D_{k}\right)=\left(\sqrt[{\alpha }]{1-{\Pi_{i=1}^{k}\left(1-\psi_{i}^{\alpha }\right)}^{\tau_{i}}},\Pi_{i=1}^{k}\eta_{i}^{\tau_{i}},\Pi_{i=1}^{k}\theta_{i}^{\tau_{i}}\right)$$

**Step 3.** Now, k=n+1, using Equations (12), (14) we have

$$\alpha ,\beta ,\gamma -\text{NWA}\left(D_{1},D_{2},\ldots ,D_{k}\right)⊕D_{k+1}=\left(\sqrt[{\alpha }]{1-{\Pi_{i=1}^{k}\left(1-\psi_{i}^{\alpha }\right)}^{\tau_{i}}},\Pi_{i=1}^{k}\eta_{i}^{\tau_{i}},\Pi_{i=1}^{k}\theta_{i}^{\tau_{i}}\;\right)$$

$$⊕\left(\sqrt[{\alpha }]{1-{\left(1-\psi_{k+1}^{\alpha }\right)}^{\tau_{k+1}}},{\left(\eta_{k+1}\right)}^{\tau_{k+1}},{\left(\theta_{k+1}\right)}^{\tau_{k+1}}\;\right)=\left(\sqrt[{\alpha }]{1-{\Pi_{i=1}^{k+1}\left(1-\psi_{i}^{\alpha }\right)}^{\tau_{i}}},\Pi_{i=1}^{k+1}\eta_{i}^{\tau_{i}},\Pi_{i=1}^{k+1}\theta_{i}^{\tau_{i}}\;\right)$$

$$=\alpha ,\beta ,\gamma -N\text{WA}\;\left(D_{1},D_{2},\ldots .,D_{k+1}\right)\text{.}$$

Thus, the result is valid for n=k+1.

Example 2 Assume four α,β,γ− NNs $D_{1}=\left(0.77,0.50,0.30\right)$, $D_{2}=\left(0.70,0.40,0.10\right)$, $D_{3}=\left(0.55,0.35,0.20\right)$ and $D_{4}=\left(0.87,0.42,0.40\right)$ with weight vector $\omega ={\left(0.20,0.25,0.27,0.28\right)}^{T}$ where α=β=4 and γ=LCM(α,β)=4. To aggregate these values, we use the aggregation operator presented in Equation (13).

$$\alpha ,\beta ,\gamma -\text{NWA}\left(D_{1},D_{2},\ldots ,D_{4}\right)=\left(\sqrt[{\alpha }]{1-{\Pi_{i=1}^{4}\left(1-\psi_{i}^{\alpha }\right)}^{\tau_{i}}},\Pi_{i=1}^{4}\eta_{i}^{\tau_{i}},\Pi_{i=1}^{4}\theta_{i}^{\tau_{i}}\right)$$

$$\sqrt[{\alpha }]{1-{\Pi_{i=1}^{4}\left(1-\psi_{i}^{\alpha }\right)}^{\tau_{i}}}=\sqrt[{4}]{\begin{array}{c} 1-{\left(1-0.77^{4}\right)}^{0.20}{\left(1-0.70^{4}\right)}^{0.25}\\ {\left(1-0.55^{4}\right)}^{0.27}{\left(1-0.87^{4}\right)}^{0.28} \end{array}}=0.7650\text{.}$$

$$\Pi_{i=1}^{4}\eta_{i}^{\tau_{i}}=0.50^{0.20}\times 0.40^{0.25}\times 0.35^{0.27}\times 0.42^{0.28}=0.4090\text{.}$$

$$\Pi_{i=1}^{4}\theta_{i}^{\tau_{i}}=0.30^{0.20}\times 0.10^{0.25}\times 0.20^{0.27}\times 0.40^{0.28}=0.2215\text{.}$$

$$\alpha ,\beta ,\gamma -\text{NWA}\left(D_{1},D_{2},\ldots ,D_{4}\right)=\left(0.7650,0.4090,0.2215\right)\text{.}$$

### 4.2 α,β,γ−NOWA operator

Definition 14 Let be a collection of α,β,γ− NNs $D_{i}=\left(\psi_{i},\eta_{i},\theta_{i}\right)$ (i=1,2,…,n) with weight vector $\tau_{i}$ such that $\tau_{i}\in \left[0,1\right]$ and $\sum_{i=1}^{n}\tau_{i}=1$. A mapping $\alpha ,\beta ,\gamma -\text{NOWA}:\Lambda^{n}\to \Lambda$ where Λ represent the collection of α,β,γ− NN, called α,β,γ−NOWA operator and presented in Equation (15).

(15) $$\alpha ,\beta ,\gamma -\text{NOWA}\left(D_{1},D_{2},\ldots ,D_{n}\right)={⊕}_{i=1}^{n}\left(\tau_{i}D_{\sigma \left(i\right)}\right)$$

Theorem 3 *Suppose that*$D_{i}=\left(\psi_{i},\eta_{i},\theta_{i}\right)$*be the collection of*α,β,γ−*NNs for all*i=1,2,…,n. *Then the aggregation result obtained by*α,β,γ−NOWA*operator is also*α,β,γ−*NNs and given by*

(16) $$\alpha ,\beta ,\gamma -\text{NOWA}\left(D_{1},D_{2},\ldots ,D_{n}\right)=\left(\begin{array}{c} \sqrt[{\alpha }]{1-{\Pi_{i=1}^{n}\left(1-\psi_{\sigma \left(i\right)}^{\alpha }\right)}^{\tau_{i}}},\\ \Pi_{i=1}^{n}\eta_{\sigma \left(i\right)}^{\tau_{i}},\\ \Pi_{i=1}^{n}\theta_{\sigma \left(i\right)}^{\tau_{i}} \end{array}\right)$$

In Equation (16), $\tau_{i}$ is the weight vector with the condition $\sum_{i=1}^{n}\tau_{i}=1$ and p any positive integer and σ(1),σ(2),…,σ(n) such that $D_{\sigma \left(n-1\right)}\geq D_{\sigma \left(n\right)}$.

**Proof.** Straightforward.

### 4.3 α,β,γ−NWG operator

Definition 15 For any collection of α,β,γ− NNs $D_{i}=\left(\psi_{i},\eta_{i},\theta_{i}\right)$ (i=1,2,…,n) with weight vector $\tau_{i}$ such that $\tau_{i}\in \left[0,1\right]$ and $\sum_{i=1}^{n}\tau_{i}=1$. A mapping $\alpha ,\beta ,\gamma -\text{NWG}:\Lambda^{n}⟶\Lambda$ where Λ represent the collection of α,β,γ− NN, called α,β,γ−NWG operator and presented in Equation (17).

(17) $$\alpha ,\beta ,\gamma -\left(D_{1},D_{2},\ldots ,D_{n}\right)={⨂}_{i=1}^{n}\left(D_{i}^{\tau_{i}}\right)$$

Theorem 4 *Let*${Η}_{i}=\left(T_{i},I_{i},F_{i}\right)$*be a collection of*α,β,γ−*NNs* (i=1,2,…,n). *The aggregation value obtained by* α,β,γ−NWG *operator is also* α,β,γ− *NNs and can be represented as follows*:

(18) $$\alpha ,\beta ,\gamma -\text{NWG}\left(D_{1},D_{2},\ldots ,D_{n}\right)=\left(\begin{array}{c} \Pi_{i=1}^{n}\psi_{i}^{\tau_{i}},\\ \sqrt[{\gamma }]{1-{\Pi_{i=1}^{n}\left(1-\eta_{i}^{\gamma }\right)}^{\tau_{i}}},\\ \sqrt[{\beta }]{1-{\Pi_{i=1}^{n}\left(1-\theta_{i}^{\beta }\right)}^{\tau_{i}}} \end{array}\right)$$

where $\tau_{i}$ is the weight vector with the condition $\sum_{i=1}^{n}\tau_{i}=1$ such that β, γ≥1.

**Proof.** Mathematical induction is utilized to prove the validity of Theorem 4.

**Step 1.** For n=2, we have $D_{1}=\left(\psi_{1},\eta_{1},\theta_{1}\right)$ and $D_{2}$ = $\left(\psi_{2},\eta_{2},\theta_{2}\right)$.

$$\alpha ,\beta ,\gamma -\text{NWG}\left(D_{1},D_{2}\right)=\left(\psi_{1}^{\tau_{1}},\sqrt[{\gamma }]{1-{\left(1-\eta_{1}^{\gamma }\right)}^{\tau_{1}}},\sqrt[{\beta }]{1-{\left(1-\theta_{1}^{\beta }\right)}^{\tau_{1}}}\;\right)$$

$$⊗\left(\psi_{2}^{\tau_{2}},\sqrt[{\gamma }]{1-{\left(1-\eta_{2}^{\gamma }\right)}^{\tau_{2}}},\sqrt[{\beta }]{1-{\left(1-\theta_{2}^{\beta }\right)}^{\tau_{2}}}\;\right)=\left(\begin{array}{c} {\left(\psi_{1}\right)}^{\tau_{1}}{\left(\psi_{2}\right)}^{\tau_{2}},\sqrt[{\gamma }]{1-{\left(1-\eta_{1}^{\gamma }\right)}^{\tau_{1}}{\left(1-\eta_{2}^{\gamma }\right)}^{\tau_{2}}},\\ \sqrt[{\beta }]{1-{\left(1-\theta_{1}^{\beta }\right)}^{\tau_{1}}{\left(1-\theta_{2}^{\beta }\right)}^{\tau_{2}}}\; \end{array}\right)$$

$$=\left(\begin{array}{c} \Pi_{i=1}^{2}\psi_{i}^{\tau_{i}},\sqrt[{\gamma }]{1-{\Pi_{i=1}^{2}\left(1-\eta_{i}^{\gamma }\right)}^{\tau_{i}}},\\ \sqrt[{\beta }]{1-{\Pi_{i=1}^{2}\left(1-\theta_{i}^{\beta }\right)}^{\tau_{i}}} \end{array}\right)$$

Thus, the result is valid for n=2.

**Step 2.** We suppose that Equation (18) is valid for n=k, i.e.,

(19) $$\alpha ,\beta ,\gamma -\text{NWG}\left(D_{1},D_{2},\ldots ,D_{k}\right)=\left(\begin{array}{c} \Pi_{i=1}^{k}\psi_{i}^{\tau_{i}},\sqrt[{\gamma }]{1-{\Pi_{i=1}^{k}\left(1-\eta_{i}^{\gamma }\right)}^{\tau_{i}}},\\ \sqrt[{\beta }]{1-{\Pi_{i=1}^{k}\left(1-\theta_{i}^{\beta }\right)}^{\tau_{i}}} \end{array}\right)$$

**Step 3.** For n=k+1, using Equation (19) we have

$$\alpha ,\beta ,\gamma -\text{NWG}\left(D_{1},D_{2},\ldots ,D_{k}\right)⊗D_{k+1}$$

$$=\left(\begin{array}{c} \Pi_{i=1}^{k}\psi_{i}^{\tau_{i}},\sqrt[{\gamma }]{1-{\Pi_{i=1}^{k}\left(1-\eta_{i}^{\gamma }\right)}^{\tau_{i}}},\\ \sqrt[{\beta }]{1-{\Pi_{i=1}^{k}\left(1-\theta_{i}^{\beta }\right)}^{\tau_{i}}} \end{array}\right)⊗\left(\begin{array}{c} {\left(\psi_{k+1}\right)}^{\tau_{k+1}},\sqrt[{\gamma }]{1-{\left(1-\eta_{k+1}^{\gamma }\right)}^{\tau_{k+1}}},\\ \sqrt[{\beta }]{1-{\left(1-\theta_{k+1}^{\beta }\right)}^{\tau_{k+1}}} \end{array}\right)$$

$$=\left(\begin{array}{c} \Pi_{i=1}^{k+1}\psi_{i}^{\tau_{i}},\sqrt[{\gamma }]{1-{\Pi_{i=1}^{k+1}\left(1-\eta_{i}^{\gamma }\right)}^{\tau_{i}}},\\ \sqrt[{\beta }]{1-{\Pi_{i=1}^{k+1}\left(1-\theta_{i}^{\beta }\right)}^{\tau_{i}}}\; \end{array}\right)$$

$$=\alpha ,\beta ,\gamma -\text{NWG}\left(D_{1},D_{2},\ldots ,D_{k+1}\right)\text{.}$$

Thus, the result is valid for n=k+1.

Example 3 Assume four α,β,γ− NNs $D_{1}=\left(0.77,0,50,0.30\right)$, $D_{2}=\left(0.70,0.40,0.10\right)$, $D_{3}=\left(0.55,0.35,0.20\right)$ and $D_{4}=\left(0.87,0.42,0.40\right)$, the weights for these α,β,γ− NNs are $\tau {=\left(0.20,0.25,0.27,0.28\right)}^{T}$ such that α=β=4, and γ=LCM(α,β)=4. To aggregate this value, we use the equation of α,β,γ−NWG operator.

$$\alpha ,\beta ,\gamma -\text{NWG}\left(D_{1},D_{2},\ldots ,D_{4}\right)=\left(\begin{array}{c} \Pi_{i=1}^{4}\psi_{i}^{\tau_{i}},\sqrt[{\gamma }]{1-{\Pi_{i=1}^{4}\left(1-\eta_{i}^{\gamma }\right)}^{\tau_{i}}},\\ \sqrt[{\beta }]{1-{\Pi_{i=1}^{4}\left(1-\theta_{i}^{\beta }\right)}^{\tau_{i}}}\; \end{array}\right)$$

$$\Pi_{i=1}^{4}\psi_{i}^{\tau_{i}}=0.77^{0.20}\times 0.70^{0.25}\times 0.55^{0.27}\times 0.87^{0.28}=0.7150\text{.}$$

$$\sqrt[{\gamma }]{1-{\Pi_{i=1}^{4}\left(1-\eta_{i}^{\gamma }\right)}^{\tau_{i}}}=\sqrt[{4}]{\begin{array}{c} 1-{\left(1-0.50^{4}\right)}^{0.20}{\left(1-0.40^{4}\right)}^{0.25}\\ {\left(1-0.35^{4}\right)}^{0.27}{\left(1-0.42^{4}\right)}^{0.28} \end{array}}=0.4223\text{.}$$

$$\sqrt[{\beta }]{1-{\Pi_{i=1}^{4}\left(1-\theta_{i}^{\beta }\right)}^{\tau_{i}}}=\sqrt[{4}]{\begin{array}{c} 1-{\left(1-0.30^{4}\right)}^{0.20}{\left(1-0.10^{4}\right)}^{0.25}\\ {\left(1-0.20^{4}\right)}^{0.27}{\left(1-0.40^{4}\right)}^{0.28} \end{array}}=0.3106\text{.}$$

Thus, the aggregated value is $\alpha ,\beta ,\gamma -\left(D_{1},D_{2},D_{3},D_{4}\right)=\left(0.7150,0.4223,0.3106\right)$.

### 4.4 α,β,γ−NOWG operator

Definition 16 For any collection of α,β,γ− NNs $D_{i}=\left(\psi_{i},\eta_{i},\theta_{i}\right)$ for all i,(i ranging from 1 to n), having weights $\tau =\left(\tau_{1},\tau_{2},\ldots ,\tau_{n}\right)$ such that $\tau_{i}\in \left[0,1\right]$ and $\sum_{i=1}^{n}\tau_{i}=1$ respectively. Then mapping $\alpha ,\beta ,\gamma -\text{NOWG}:\Omega^{n}\to \Omega$ where Ω represent the group of α,β,γ− NNs, called α,β,γ−NOWG operator and presented in Equation (20).

(20) $$\alpha ,\beta ,\gamma -\text{NOWG}\left(D_{1},D_{2},\ldots ,D_{n}\right)={⊗}_{i=1}^{n}\left(D_{\sigma \left(i\right)}^{\tau_{i}}\right)$$

Theorem 5 *Suppose that*$D_{i}=\left(\psi_{i},\eta_{i},\theta_{i}\right)$*be the collection of*α,β,γ−*NNs for all*i (i *ranges from* 1 *to* n). *Then the aggregate result obtained by* α,β,γ−NOWG *operator is also* α,β,γ− *NN and can be expressed as follows*:

(21) $$\alpha ,\beta ,\gamma -\left(D_{1},D_{2},\ldots ,D_{n}\right)=\left(\begin{array}{c} \Pi_{i=1}^{n}\psi_{\sigma \left(i\right)}^{\tau_{i}},\\ \sqrt[{\gamma }]{1-{\Pi_{i=1}^{n}\left(1-\eta_{\sigma \left(i\right)}^{\gamma }\right)}^{\tau_{i}}},\\ \sqrt[{\beta }]{1-{\Pi_{i=1}^{n}\left(1-\theta_{\sigma \left(i\right)}^{\beta }\right)}^{\tau_{i}}} \end{array}\right)$$

In Equation (21), $\tau_{i}$ (i=1,2,…,n) are the weights for α,β,γ− NNs with the conditions $\tau_{i}\in \left[0,1\right]$ and $\sum_{i=1}^{n}\tau_{i}=1$ such that α,β≻0 and γ=(α,β), where $D_{\sigma \left(n-1\right)}≽{Η}_{\sigma \left(n\right)}$.

**Proof:** The prove is similar to Theorem 4.

### 4.5 Properties of proposed AOs

Property 1 $\alpha ,\beta ,\gamma -\text{NWA}\left(D_{1},D_{2},\ldots ,D_{n}\right)=D$, if all α,β,γ− NNs $D_{i}=D$ where D represent the another α,β,γ− NNs.

**Proof.** Let D(ψ,η,θ) and $D_{i}$ = D for all i=1,2,…,n, then we have

$$\Rightarrow \;\alpha ,\beta ,\gamma -\left(D_{1},D_{2},\ldots ,D_{n}\right)=\left(\sqrt[{\alpha }]{1-{\Pi_{i=1}^{n}\left(1-\psi_{i}^{\alpha }\right)}^{\tau_{i}}},\Pi_{i=1}^{n}\eta_{i}^{\tau_{i}},\Pi_{i=1}^{n}\theta_{i}^{\omega_{i}}\;\right)$$

$$\Rightarrow \sqrt[{\alpha }]{1-{\Pi_{i=1}^{n}\left(1-\psi_{i}^{\alpha }\right)}^{\tau_{i}}}=\sqrt[{\alpha }]{1-{\Pi_{i=1}^{n}\left(1-\psi^{\alpha }\right)}^{\sum_{i=1}^{n}\tau_{i}}}$$

$$=\sqrt[{\alpha }]{1-\left(1-\psi^{\alpha }\right)}=\sqrt[{\alpha }]{T^{\alpha }}=\psi \text{.}$$

$$\Pi_{i=1}^{n}\eta_{i}^{\tau_{i}}=\eta^{\tau_{1}}\times \eta^{\tau_{2}}\times \ldots \times \eta^{\tau_{n}}=\eta^{\sum_{i=1}^{n}\tau_{i}}=\eta \text{.}$$

$$\Pi_{i=1}^{n}\theta_{i}^{\tau_{i}}=\theta^{\tau_{1}}\times \theta^{\tau_{2}}\times \ldots \times \theta^{\tau_{n}}=\theta^{\sum_{i=1}^{n}\tau_{i}}=\theta \text{.}$$

$$\alpha ,\beta ,\gamma -\left(D_{1},D_{2},\ldots ,D_{n}\right)=\left(\psi ,\eta ,\theta \right)=D\text{.}$$

Similarly, $\alpha ,\beta ,\gamma -\text{NOWA}\left(D_{1},D_{2},\ldots ,D_{n}\right)=D$, if all α,β,γ− NNs $D_{i}=D$ where D represent the another α,β,γ− NNs.

$\alpha ,\beta ,\gamma -\text{NWG}\left(D_{1},D_{2},\ldots ,D_{n}\right)=D$, if all α,β,γ− NNs $D_{i}=D$ where D represent the another α,β,γ− NNs.

$\alpha ,\beta ,\gamma -\text{NOWG}\left(D_{1},D_{2},\ldots ,D_{n}\right)=D$, if all α,β,γ− NNs $D_{i}=D$ where D represent the another α,β,γ− NNs.

Property 2 *Let*$D^{-}=\left\langle \psi_{\min},\eta_{\max},\theta_{\max}\right\rangle$*and*$D^{+}=\left\langle \psi_{\max},\eta_{\min},\theta_{\min}\right\rangle$, *then*

a. $D^{-}\leq \alpha ,\beta ,\gamma -\text{NWA}\left(D_{1},D_{2},\ldots ,D_{n}\right)\leq D^{+}$,
b. $D^{-}\leq \alpha ,\beta ,\gamma -\text{NOWA}\left(D_{1},D_{2},\ldots ,D_{n}\right)\leq D^{+}$,
c. $D^{-}\leq \alpha ,\beta ,\gamma -\text{NWG}\left(D_{1},D_{2},\ldots ,D_{n}\right)\leq D^{+}$,
d. $D^{-}\leq \alpha ,\beta ,\gamma -\text{NOWG}\left(D_{1},D_{2},\ldots ,D_{n}\right)\leq D^{+}$.

Property 3 *Consider the collection of two of*α,β,γ−*NNs*$D_{i}=$ ($\psi_{i},\eta_{i},\theta_{i}$) *and* $D_{i}^{*}$ = ($\psi_{i}^{*},\eta_{i}^{*},\theta_{i}^{*}$) *such that* $\psi_{i}\leq \psi_{i}^{*},\eta_{i}\geq \eta_{i}^{*}$, $\theta_{i}\geq \theta_{i}^{*}$, *then*

a. $\alpha ,\beta ,\gamma -\text{NWA}\left(D_{1},D_{2},\ldots ,D_{n}\right)≼\alpha ,\beta ,\gamma -N\text{WA}\left(D_{1}^{*},D_{2}^{*},\ldots ,D_{n}^{*}\right)$,
b. $\alpha ,\beta ,\gamma -\text{NOWA}\left(D_{1},D_{2},\ldots ,D_{n}\right)≼\alpha ,\beta ,\gamma -\text{NOWA}\left(D_{1}^{*},D_{2}^{*},\ldots ,D_{n}^{*}\right)$,
c. $\alpha ,\beta ,\gamma -\text{NWG}\left(D_{1},D_{2},\ldots ,D_{n}\right)≼\alpha ,\beta ,\gamma -N\text{WG}\left(D_{1}^{*},D_{2}^{*},\ldots ,D_{n}^{*}\right)$,
d. $\alpha ,\beta ,\gamma -\text{NOWG}\left(D_{1},D_{2},\ldots ,D_{n}\right)≼\alpha ,\beta ,\gamma -\text{NOWG}\left(D_{1}^{*},D_{2}^{*},\ldots ,D_{n}^{*}\right)$,

Property 4 *if*$\left(D_{1}^{⋆},D_{2}^{⋆},\ldots ,D_{n}^{⋆}\right)$*be any permutation of*α,β,γ−*NNs in*$\left(D_{1},D_{2},\ldots .,D_{n}\right)$, *then*

a. $\alpha ,\beta ,\gamma -N\text{WA}\left(D_{1},D_{2},\ldots ,D_{n}\right)=\alpha ,\beta ,\gamma -\text{NWA}\left(D_{1}^{⋆},D_{2}^{⋆},\ldots .,D_{n}^{⋆}\right)$,
b. $\alpha ,\beta ,\gamma -\text{NOWA}\left(D_{1},D_{2},\ldots ,D_{n}\right)=\alpha ,\beta ,\gamma -\text{NOWA}\left(D_{1}^{⋆},D_{2}^{⋆},\ldots .,D_{n}^{⋆}\right)$,
c. $\alpha ,\beta ,\gamma -N\text{WG}\left(D_{1},D_{2},\ldots ,D_{n}\right)=\alpha ,\beta ,\gamma -\text{NWG}\left(D_{1}^{⋆},D_{2}^{⋆},\ldots .,D_{n}^{⋆}\right)$,
d. $\alpha ,\beta ,\gamma -\text{NOWG}\left(D_{1},D_{2},\ldots ,D_{n}\right)=\alpha ,\beta ,\gamma -\text{NOWG}\left(D_{1}^{⋆},D_{2}^{⋆},\ldots .,D_{n}^{⋆}\right)$.

Property 5 *If* ξ≽0 *then*

a. $\alpha ,\beta ,\gamma -\text{NWA}\left({\xi D}_{1},{\xi D}_{2},\ldots ,{\xi D}_{n}\right)=\alpha ,\beta ,\gamma -\xi N\text{WA}\left(D_{1},D_{2},\ldots ,D_{n}\right)$,
b. $\alpha ,\beta ,\gamma -\text{NOWA}\left({\xi D}_{1},{\xi D}_{2},\ldots ,{\xi D}_{n}\right)=\alpha ,\beta ,\gamma -\xi \text{NOWA}\left(D_{1},D_{2},\ldots ,D_{n}\right)$,
c. $\alpha ,\beta ,\gamma -\text{NWG}\left({\xi D}_{1},{\xi D}_{2},\ldots ,{\xi D}_{n}\right)=\alpha ,\beta ,\gamma -\xi N\text{WG}\left(D_{1},D_{2},\ldots ,D_{n}\right)$,
d. $\alpha ,\beta ,\gamma -\text{NOWG}\left({\xi D}_{1},{\xi D}_{2},\ldots ,{\xi D}_{n}\right)=\alpha ,\beta ,\gamma -\xi \text{NOWG}\left(D_{1},D_{2},\ldots ,D_{n}\right)$.

## 5 Proposed MCDM approach

DM is a complex reasoning procedure intrinsic to various facets of life, encompassing individual choices and intricate organizational strategies alike. This multifaceted process involves the meticulous selection of a course of action from a myriad of alternatives, requiring a judicious assessment of available information, personal preferences, and situational constraints. A consciousness of the moral implications decisions ensures not only their effectiveness but also their broader societal responsibility. Ultimately, decision-making emerges as a skill that evolves through experiential learning, drawing insights from both successful and unsuccessful outcomes. Whether navigating personal junctures or steering the course of a professional trajectory, individuals and organizations stand to benefit significantly from cultivating a structured and thoughtful approach to decision-making, recognizing it as a dynamic and indispensable facet of human cognition and interaction.

The introduced DM approach depend on novel aggregation operators with a parametric nature, offering decision makers an elastic framework. The parametric features of these operators establish an adaptable environment, allowing decision makers to tailor the approach to their specific needs. The parameters incorporated into this DM framework play a pivotal role in governing the impact of membership degrees associated with information related to various alternatives. In essence, these parameters provide a mechanism for decision makers to regulate and fine-tune the influence of information membership degrees, enabling a nuanced and customized control over the decision-making process. This level of parameterization enhances the versatility of the approach, empowering decision makers to navigate the complexity of decision scenarios with a heightened degree of precision and responsiveness to the nuances of the available information. In formulating the foundation of the proposed approach, certain assumptions are considered.

**Fig. 2:**
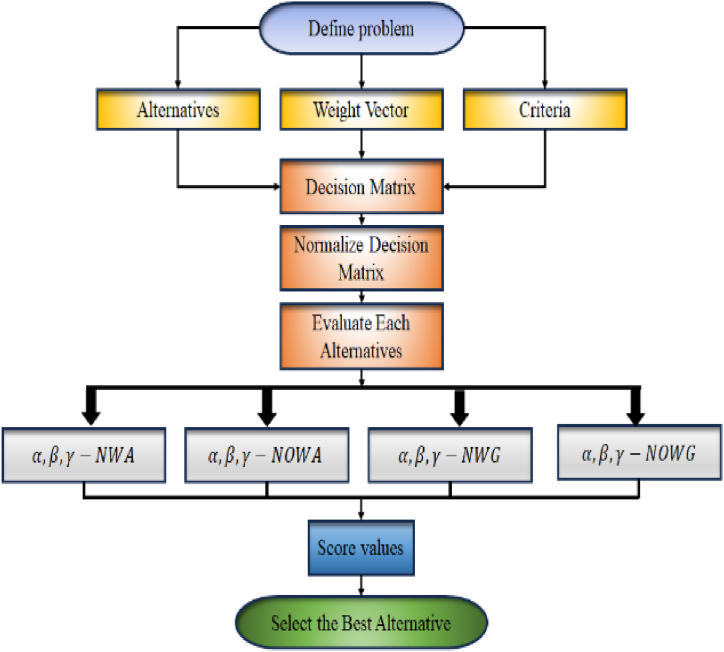
Layout of the proposed approach.

Let $Y=\left\{Y_{1},Y_{2},\ldots ,Y_{m}\right\}$ be a set of m alternatives, that requires analysis within the context of a set of n diverse criteria, represented by $X=\left\{X_{1},X_{2},\ldots ,X_{n}\right\}$. Consider the scenario where a set of m distinct alternatives, undergoes evaluation by experts who express their preferences for each alternative $Y_{i}$ (where i ranges from 1 to m) within the context of the α,β,γ− NSs environment. These evaluations result in values that can be represented as α,β,γ− NNs $D={\left(d_{\text{ij}}\right)}_{m\times n}$, where $d_{\text{ij}}=\left(\psi_{\text{ij}},\eta_{\text{ij}},\theta_{\text{ij}}\right)$, signifying the priority values assigned to alternatives $Y_{i}$ by the decision maker. Here, $\psi_{\text{ij}}$, $\eta_{\text{ij}}$ and $\theta_{\text{ij}}\in \left[0,1\right]$, with the constraints $\psi_{\text{ij}}^{\alpha }+\eta_{\text{ij}}^{\beta }+\theta_{\text{ij}}^{\gamma }≼3$ for all i=1,2,…,m and j=1,2,…,n. Let $\tau =\left(\tau_{1},\tau_{2},\ldots ,\tau_{n}\right)$ be the weight vector of the criteria, where $0≼\tau_{j}≼1$ and $\sum_{n=1}^{n}\tau_{j}=1$. The proposed method is outlined in several steps designed to identify the optimal alternative in this complex decision-making context. The layout of the presented approach is listed in Fig. 2.

Step 1. Gather the information ratings for the alternatives based on the criteria and condense them into the form of a α,β,γ− NNs, represented as $d_{\text{ij}}=\left(\psi_{\text{ij}},\eta_{\text{ij}},\theta_{\text{ij}}\right)$. These rating values are articulated within a decision matric D as presented in Equation (22).

(22) $$D=\left(\begin{array}{ccc} d_{11} & \cdots & d_{1n}\\ \vdots & \ddots & \vdots \\ d_{m1} & \cdots & d_{\text{mn}} \end{array}\right)$$

Step 2. DM involves evaluating criteria that are generally characterized into cost and benefit criteria. Cost criteria typically involve factors where lower values are preferable, such as minimizing expenses, resource usage, or time requirements. On the contrary, benefit criteria encompass aspects where higher values are desirable, such as revenue generation, customer satisfaction, or project efficiency. The interplay between these two types of criteria is pivotal in achieving well-rounded decisions, as decision-makers aim to strike a balance between minimizing costs and maximizing benefits. Analytical tools like cost-benefit analysis assist in quantifying and comparing the impact of different criteria, facilitating a comprehensive evaluation of alternatives and guiding the selection of optimal solutions that align with overarching goals and objectives. Standardize the cooperative information decision matrix by translating the rating values of cost type ($B_{j}$) into benefit type ($C_{j}$), if applicable, through the utilization of the normalization formula:

(23) $$n_{\text{ij}}=\left\{ \begin{array}{c} \left(\psi_{\text{ij}},\eta_{\text{ij}},\theta_{\text{ij}}\right);\;B_{j}\\ \left(\theta_{\text{ij}},\eta_{\text{ij}},\psi_{\text{ij}}\right);\;C_{j} \end{array}\right.$$

Step 3. Utilize the proposed aggregation operators to aggregate the information related to the alternatives.
Step 4. Compute score values using the formula stated in Equation (24).

(24) $$\text{Sc}\left(n_{\text{ij}}\right)=\frac{2+{\left(\psi_{\text{ij}}\right)}^{\alpha }-{\left(\eta_{\text{ij}}\right)}^{\;\beta }-{\left(\theta_{\text{ij}}\right)}^{\gamma }}{3}$$

where $\psi_{\text{ij}}$, $\eta_{\text{ij}}$, $\theta_{\text{ij}}\in \left[0,1\right]$ and α, β and γ are positive integers such that $\psi_{\text{ij}}^{\alpha }+\eta_{\text{ij}}^{\beta }+\theta_{\text{ij}}^{\gamma }≼3$. The incorporation of these functions is designed to generate exclusively positive outcomes by adding 2 with ${\left(\psi_{\text{ij}}\right)}^{\alpha }-{\left(\eta_{\text{ij}}\right)}^{\;\beta }-{\left(\theta_{\text{ij}}\right)}^{\gamma }$. Following this, all obtained values are then divided by 3, effectively limiting the range of score values to the interval [0,1].

Step 5. Alternative rank the alternatives according to the score values.

### 5.1 Numerical example

Within this section, we present an illustration showcasing the application of our proposed model in the process of determining the optimal location for a software office.

Assume that a multinational company (MNC) want to establish a software office, to enhance business profits while minimize costs. In pursuit of this goal, the company is considering four different countries, denoted as $Y_{1}$, $Y_{2}$, $Y_{3}$ and $Y_{4}$. For the smooth operation of their software business, they require a country that is not only economically robust but also offers all essential infrastructure, facilities, and cost-effective solutions for transportation and labor. To make an informed decision, the company's decision-making committee, represented by the weight vector $\tau ={\left(0.20,0.25,0.27,0.28\right)}^{T}$, has identified specific attributes based on their priorities to select the most suitable country. The attributes are as follows:

Proximity to the main market ($X_{1}$): The company, being well-established with a global market presence, aims to enhance communication by minimizing the distance between their operational center and the primary location of their main customers.

Routine cost ($X_{2}$): To ensure the seamless operation of their software unit, the company seeks a location where expenses associated with transportation, energy, tool acquisition, and both skilled and unskilled labor costs are minimized.

Facilities providing by the government of that country ($X_{3}$): The local government needs to offer resources such as ample space, efficient transportation, and reliable electricity to MNC. Therefore, the company should prefer a country that demonstrates greater cooperation from the government, as the availability of these resources can be vary across different countries.

Accessibility of both skilled and new employees ($X_{4}$): To ensure the efficient operation of the software unit, the company requires a significant workforce, comprising both skilled and unskilled labor. The company prioritizes countries with access to skilled engineers and unskilled workers at the lowest possible cost to optimize expenditures.

**Fig. 3:**
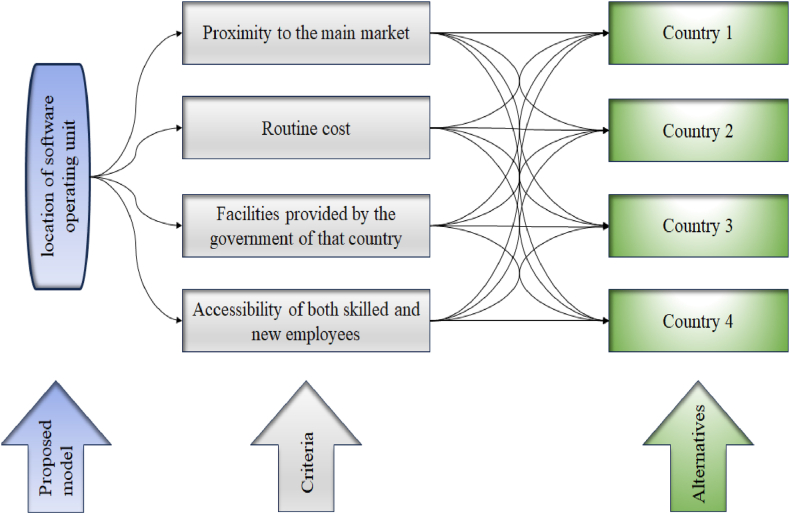
Proposed model.

Now, selecting the optimal location for setting up the software unit is a complex and uncertain decision, given the lack of information and conflicting factors. To address challenge, we employed α,β,γ− NSs as a valuable tool, considering their broader perspectives. Consequently, the management committee has articulated their preferences for the alternatives in terms of α,β,γ− NNs. The layout of the proposed model is presented in Fig. 3.

**Fig. 4:**
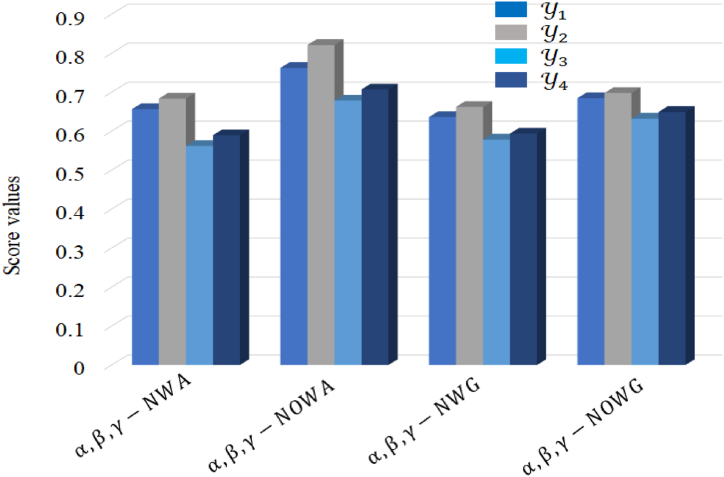
Graphical representation of score values by using the proposed AOs.

Fine-tuning parameters α, β, and γ within the proposed aggregation operators involves a meticulous assessment of the significance and ambiguity inherent in various facets of the problem domain (see Fig. 4). Through the manipulation of these parameters, decision-makers wield control over the impact of membership, non-membership, and indeterminacy degrees, consequently molding the overarching evaluation or decision-making procedure. At this juncture, a spectrum of values for α, β, and γ is explored, enabling adjustment based on contextual demands and considerations.

**Step 1.** The comprehensive data pertaining to each criterion for all alternatives is systematically presented in Table 2, offering a consolidated view of the collective information associated with each alternative.

**Table 2:** Information of alternatives $Y_{i}$ in the context of criteria $X_{j}$.

$Y_{i}$	$X_{1}$	$X_{2}$	$X_{3}$	$X_{4}$
$Y_{1}$	$\left(\begin{array}{c} 0.81,0.65\\ ,0.49 \end{array}\right)$	$\left(\begin{array}{c} 0.91,0.75\text{,}\\ 0.59 \end{array}\right)$	$\left(\begin{array}{c} 0.71,0.55\text{,}\\ 0.39 \end{array}\right)$	$\left(\begin{array}{c} 0.81,0.68\text{,}\\ 0.55 \end{array}\right)$
$Y_{2}$	$\left(\begin{array}{c} 0.91,0.70\text{,}\\ 0.49 \end{array}\right)$	$\left(\begin{array}{c} 0.21,0.50\text{,}\\ 0.79 \end{array}\right)$	$\left(\begin{array}{c} 0.89,0.69\text{,}\\ 0.49 \end{array}\right)$	$\left(\begin{array}{c} 0.51,0.45\text{,}\\ 0.39 \end{array}\right)$
$Y_{3}$	$\left(\begin{array}{c} 0.51,0.50\text{,}\\ 0.49 \end{array}\right)$	$\left(\begin{array}{c} 0.71,0.65\text{,}\\ 0.59 \end{array}\right)$	$\left(\begin{array}{c} 0.66,0.55\text{,}\\ 0.44 \end{array}\right)$	$\left(\begin{array}{c} 0.65,0.57\text{,}\\ 0.49 \end{array}\right)$
$Y_{4}$	$\left(\begin{array}{c} 0.45,0.40\text{,}\\ 0.35 \end{array}\right)$	$\left(\begin{array}{c} 0.61,0.55\text{,}\\ 0.49 \end{array}\right)$	$\left(\begin{array}{c} 0.47,0.39\text{,}\\ 0.31 \end{array}\right)$	$\left(\begin{array}{c} 0.70,0.45\text{,}\\ 0.20 \end{array}\right)$
**Step 2.** By utilizing Equation (23) to normalize the provided information. After, the normalization, we information are listed in Table 3.

**Table 3:** Normalize decision matrix.

$Y_{i}$	$X_{1}$	$X_{2}$	$X_{3}$	$X_{4}$
$Y_{1}$	$\left(\begin{array}{c} 0.81,0.65\text{,}\\ 0.49 \end{array}\right)$	$\left(\begin{array}{c} 0.59,0.75\text{,}\\ 0.91 \end{array}\right)$	$\left(\begin{array}{c} 0.71,0.55\text{,}\\ 0.39 \end{array}\right)$	$\left(\begin{array}{c} 0.81,0.68\text{,}\\ 0.55 \end{array}\right)$
$Y_{2}$	$\left(\begin{array}{c} 0.91,0.70\text{,}\\ 0.49 \end{array}\right)$	$\left(\begin{array}{c} 0.79,0.50\text{,}\\ 0.21 \end{array}\right)$	$\left(\begin{array}{c} 0.89,0.69\text{,}\\ 0.49 \end{array}\right)$	$\left(\begin{array}{c} 0.51,0.45\text{,}\\ 0.39 \end{array}\right)$
$Y_{3}$	$\left(\begin{array}{c} 0.51,0.50\text{,}\\ 0.49 \end{array}\right)$	$\left(\begin{array}{c} 0.59,0.65\text{,}\\ 0.71 \end{array}\right)$	$\left(\begin{array}{c} 0.66,0.55\text{,}\\ 0.44 \end{array}\right)$	$\left(\begin{array}{c} 0.65,0.57\text{,}\\ 0.49 \end{array}\right)$
$Y_{4}$	$\left(\begin{array}{c} 0.45,0.40\text{,}\\ 0.35 \end{array}\right)$	$\left(\begin{array}{c} 0.49,0.55\text{,}\\ 0.61 \end{array}\right)$	$\left(\begin{array}{c} 0.47,0.39\text{,}\\ 0.31 \end{array}\right)$	$\left(\begin{array}{c} 0.70,0.45\text{,}\\ 0.20 \end{array}\right)$
**Step 3.** To aggregate the information, we used the proposed AOs for α=β=γ=3. The results are summarized in Table 4, Table 5, Table 6, Table 7.

**Table 4:** Combined values of alternatives by using α,β,γ−NWA operator.

Alternatives	Aggregated values
$Y_{1}$	(0.7476,0.6522,0.5555)
$Y_{2}$	(0.8239,0.5664,0.3719)
$Y_{3}$	(0.6168,0.5683,0.5222)
$Y_{4}$	(0.5639,0.4446,0.3327)

**Table 5:** Combined values of alternatives by using α,β,γ−NOWA operator.

Alternatives	Aggregated values
$Y_{1}$	(0.7611,0.6209,0.5319)
$Y_{2}$	(0.8736,0.5442,0.3485)
$Y_{3}$	(0.6413,0.5361,0.4974)
$Y_{4}$	(0.5905,0.4311,0.3178)

**Table 6:** Combined values of alternatives by using α,β,γ−NWG operator.

Alternatives	Aggregated values
$Y_{1}$	(0.5520,0.6631,0.5747)
$Y_{2}$	(0.6686,0.5890,0.3901)
$Y_{3}$	(0.4373,0.5753,0.5406)
$Y_{4}$	(0.3810,0.4792,0.3663)

**Table 7:** Combined values of alternatives by using α,β,γ−NOWG operator.

Alternatives	Aggregated values
$Y_{1}$	(0.5923,0.6445,0.5557)
$Y_{2}$	(0.7091,0.5698,0.3712)
$Y_{3}$	(0.4781,0.5563,0.5217)
$Y_{4}$	(0.4424,0.4599,0.3471)
**Step 4.** The score values resulting from the aggregation using equation (24) are succinctly summarized in Table 8.

**Table 8:** Score values and ranking order of alternatives.

Aggregation operators	Score values				Best alternative
	$Y_{1}$	$Y_{2}$	$Y_{3}$	$Y_{4}$	
α,β,γ−NWA	0.6563	0.7621	0.6362	0.6848	$Y_{2}$
α,β,γ−NOWA	0.6836	0.8210	0.6622	0.6978	$Y_{2}$
α,β,γ−NWG	0.5622	0.6783	0.5784	0.6320	$Y_{2}$
α,β,γ−NOWG	0.5894	0.7068	0.5937	0.6491	$Y_{2}$
**Step 5.** The tabulated information in Table 8 reveals the ranking order of alternatives based on their respective score values: $\text{Sc}\left(Y_{2}\right)$ surpasses $\text{Sc}\left(Y_{4}\right)$, $\text{Sc}\left(Y_{3}\right)$, and $\text{Sc}\left(Y_{1}\right)$. Consequently, alternative a emerges as the optimal choice for the software operating unit.

### 5.2 Sensitivity analysis

In this section, we delve into an investigation of the consistency and influence of different parameter values, namely α, β, and γ, on the outcomes of the DM process. In the initial phase, our focus centers on scrutinizing the influence of the parameter α on the scored values of the alternatives. For this analysis, we employ the α,β,γ− NWA operator while keeping the value of β constant at 4. The resulting scored values for alternatives under various α values are meticulously detailed in Table 9, shedding light on the nuanced relationship between parameter variations and decision outcomes.

**Table 9:** Score values of alternatives for different values of α.

α	β	γ	$\mathrm{Sc}\left(Y_{1}\right)$	$\mathrm{Sc}\left(Y_{2}\right)$	$\mathrm{Sc}\left(Y_{3}\right)$	$\mathrm{Sc}\left(Y_{4}\right)$	**Best alternative**
1	4	4	0.6132	0.7441	0.5956	0.6625	$Y_{2}$
2	4	4	0.6245	0.7562	0.6042	0.6731	$Y_{2}$
3	4	12	0.6324	0.7681	0.6137	0.6825	$Y_{2}$
4	4	4	0.6416	0.7789	0.6228	0.6912	$Y_{2}$
5	4	20	0.6502	0.7850	0.6311	0.6997	$Y_{2}$
6	4	12	0.6591	0.7943	0.6403	0.7074	$Y_{2}$
7	4	28	0.6668	0.8034	0.6481	0.7148	$Y_{2}$
8	4	8	0.6751	0.8085	0.6576	0.7180	$Y_{2}$
9	4	36	0.6782	0.8127	0.6649	0.7219	$Y_{2}$
10	4	20	0.6793	0.8159	0.6691	0.7235	$Y_{2}$

**Fig. 5:**
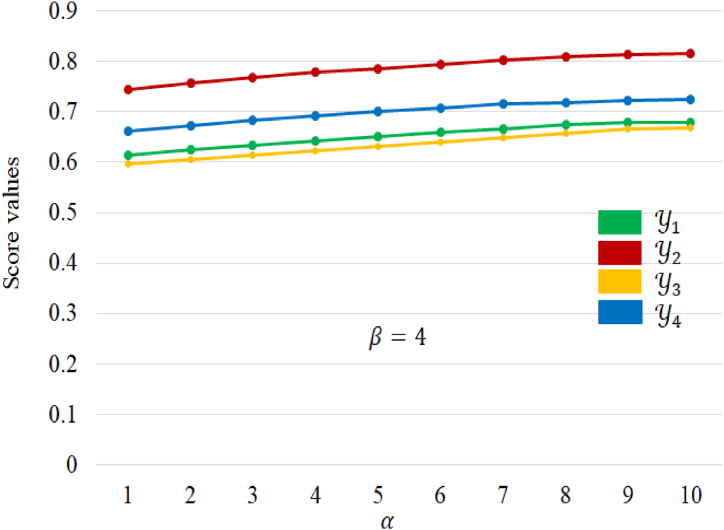
The behaver of score values with respect to the parameter α.

Upon examining the data presented in Table 9, a discernible pattern emerges: as the parameter α increases, so do the score values of the alternatives. This observation underscores the symmetry of the proposed aggregation α,β,γ− NWA operator concerning the parameter α. Notably, the ranking orders of the alternatives remain consistent despite variations in the paired parameters α and β (where β is held constant at 4), demonstrating a robust stability in decision outcomes. This particular characteristic of the α,β,γ− NWA operator holds significant implications for real decision-making scenarios. Notably, it is observed that an escalation in the parameter α corresponds to an increase in the score values of the alternatives. This optimistic trend provides decision-makers with a positive perspective, suggesting that higher values can be judiciously assigned to parameter α during the aggregation process, aligning with an optimistic stance in DM scenarios. The behaver of alternatives for different values of α is presented in Fig. 5.

On the other hand, when engaging the α,β,γ− NWG operator and analytically adjusting the parameter β across the range of 1 to 10, the resultant findings are succinctly consolidated in Table 10. This analysis sheds light on the dynamic impact of varying the parameter β within this operator, providing a comprehensive overview of how alterations in β influence the overall outcomes.

**Table 10:** Score values for different values of β (Fixed α=4).

α	β	γ	$\mathrm{Sc}\left(Y_{1}\right)$	$\mathrm{Sc}\left(Y_{2}\right)$	$\mathrm{Sc}\left(Y_{3}\right)$	$\mathrm{Sc}\left(Y_{4}\right)$	**Ranking order**
4	1	4	0.5873	0.7136	0.6074	0.6591	$Y_{2}≻Y_{4}≻Y_{3}≻Y_{1}$
4	2	3	0.5802	0.7015	0.6013	0.6414	
4	3	12	0.5728	0.6907	0.5977	0.6339	
4	4	4	0.5645	0.6827	0.5921	0.6272	
4	5	20	0.5567	0.6748	0.5884	0.6230	
4	6	12	0.5504	0.6675	0.5840	0.6191	
4	7	28	0.5472	0.6613	0.5807	0.6164	
4	8	8	0.5434	0.6549	0.5786	0.6122	
4	9	36	0.5410	0.6501	0.5754	0.6095	
4	10	20	0.5391	0.6483	0.5717	0.6082	

**Fig. 6:**
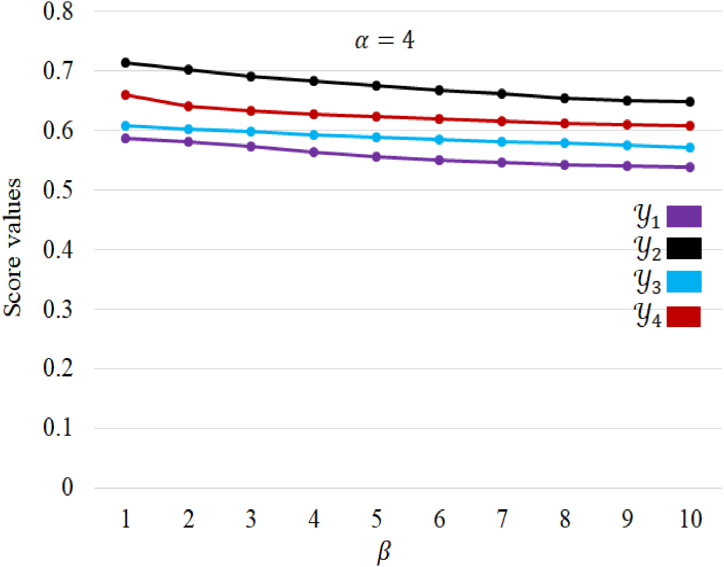
The influence of β over score values.

Examining Table 10 reveals that as the parameter β is increased, the corresponding score values exhibit a decrease. In cases where decision-makers adopt a pessimistic outlook, assigning higher values to the parameter β, the overall scores decrease. Interestingly, despite these shifts, the best alternative remains consistent. This consistency indicates that the results are objective and not subject to alteration based on the decision-makers' pessimistic or optimistic preferences. Consequently, the ranking results can be deemed reliable and robust, unaffected by individual attitudes toward pessimism or optimism. The behaver of alternatives with respect to the parameter β is presented in Fig. 6.

### 5.3 Comparative study

**Fig. 7:**
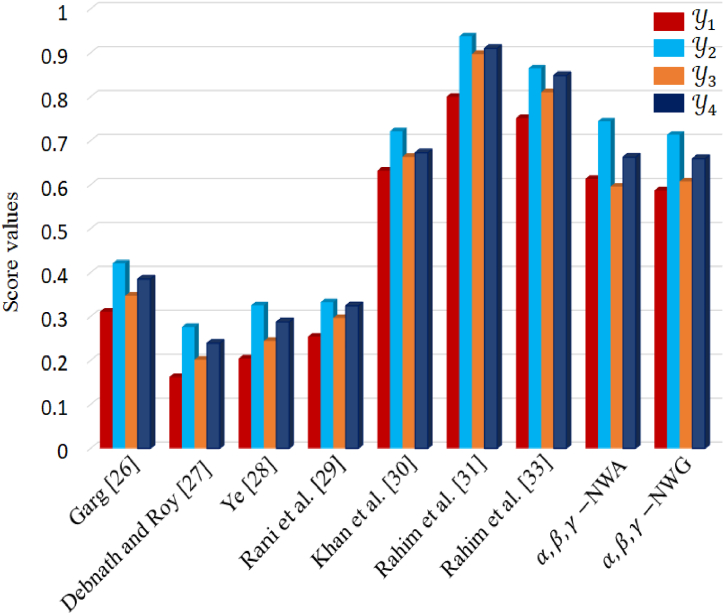
Show the comparison analysis of proposed approach with some existing approaches.

To comprehensively assess the effectiveness of our proposed model, we conduct a comparative analysis with existing approaches, namely Garg [37], Debnath and Roy [38], Ye [39] Rani et al. [40], Khan et al. [41], Rahim et al. [42] and Rahim et al. [43]. Our primary objective in this section is to establish the authenticity and superiority of our proposed model. Through a meticulous evaluation process, we compare the ranking order of alternatives generated by our model with those of the existing approaches. Notably, the alignment of the ranking order, with the same best alternative ($Y_{2}$), underscores the superior flexibility and comprehensiveness of our proposed technique. This distinction arises from the incorporation of three pivotal parameters, namely α, β, and γ, which significantly influence the evaluation of alternatives. Unlike existing approaches, our model demonstrates enhanced flexibility and a liberated structure, empowering decision-makers to navigate the complexities of the decision-making process in a more realistic manner. This flexibility is particularly evident in Table 11 and Fig. 7, where our proposed method overcomes certain limitations inherent in existing approaches.

**Table 11:** Show comparative analysis with existing approaches.

Approaches	Score values				Best Choice
	$Y_{1}$	$Y_{2}$	$Y_{3}$	$Y_{4}$	
Garg [37]	0.3113	0.4211	0.3472	0.3853	$Y_{2}$
Debnath and Roy [38]	0.1625	0.2763	0.2018	0.2394	$Y_{2}$
Ye [39]	0.2045	0.3256	0.2444	0.2878	$Y_{2}$
Rani et al. [40]	0.2542	0.3327	0.2965	0.3244	$Y_{2}$
Khan et al. [41]	0.6321	0.7216	0.6635	0.6726	$Y_{2}$
Rahim et al. [42]	0.8001	0.9376	0.8972	0.9101	$Y_{2}$
Rahim et al. [43]	0.7516	0.8648	0.8104	0.8484	$Y_{2}$
α,β,γ− NWA	0.6132	0.7441	0.5956	0.6625	$Y_{2}$
α,β,γ− NWG	0.5873	0.7136	0.6074	0.6591	$Y_{2}$

As a result, neutrosophic sets offer a general framework for representing uncertainty, α,β,γ− neutrosophic sets provide a more advanced and customizable approach by introducing parameters for fine-tuning the influence of truth-membership, indeterminacy-membership, and falsity-membership degrees. This additional level of control enhances the flexibility and effectiveness of α,β,γ− neutrosophic sets in handling uncertainty in decision-making processes.

### 5.4 Advantages

Some advantages of the proposed as follows:

• Fine-tuning Membership Influence

In traditional neutrosophic sets, the influence of membership degrees remains fixed, providing limited control over the importance assigned to elements confidently belonging to a set. However, in the α,β,γ− neutrosophic set framework, the parameter α offers the ability to adjust and regulate the influence of membership degrees. As α increases, the influence of membership degrees decreases, allowing decision-makers to downplay the importance of elements confidently categorized within a set.

• Tailored Decision Context

The parameter α enables decision-makers to tailor the influence of membership degrees according to the specific context of the decision-making process. By adjusting α based on factors such as the reliability of data, the confidence level in information sources, or the nature of the decision problem, decision-makers can effectively regulate the impact of membership degrees to better align with the decision context.

• Balancing Certainty and Uncertainty

α,β,γ− neutrosophic sets allow decision-makers to strike a balance between certainty and uncertainty in the decision-making process. By decreasing the influence of membership degrees through α, β and γ, decision-makers can account for the inherent uncertainty and ambiguity in real-world data and information, ensuring that decisions are not overly reliant on confidently categorized elements but instead consider a broader spectrum of possibilities.

• Adaptive Decision Strategies

The flexibility provided by the parameter α allows decision-makers to adopt adaptive decision strategies that respond to changing conditions or evolving information during the decision-making process. By dynamically adjusting α, β and γ based on the evolving level of certainty or confidence in the available data and information, decision-makers can maintain agility and responsiveness in their decision-making approach.

• Enhanced Robustness and Reliability

Effectively regulating the influence of membership degrees through α, β and γ, α,β,γ− neutrosophic sets contributes to the development of more robust and reliable decision-making processes. Decision-makers can mitigate the risks associated with over-reliance on confidently categorized elements by incorporating a more balanced consideration of uncertainty and ambiguity, leading to decisions that are more resilient to unforeseen contingencies or variations in the decision environment.

### 5.5 Limitations

Some limitations of the proposed works as follows.

1. The article emphasizes a case study for selecting an optimal software office location to demonstrate the flexibility and reliability of the proposed model. However, the scope of validation may be limited to this specific scenario. The study could benefit from exploring a broader range of real-life decision-making challenges across different industries to ensure the generalizability and robustness of the α,β,γ− NS framework.
2. The introduction of parameters α, β, and γ in the α,β,γ-NSs framework is a significant innovation. However, the study may not thoroughly investigate the sensitivity of the proposed model to variations in these parameters. Understanding how changes in α, β, and γ values impact the results and ensuring the model's stability under different conditions is crucial for practical implementation. Calibration guidelines or sensitivity analyses could enhance the model's reliability.

## 6 Conclusion

In this article, we have developed an extensive framework for MCDM approach that is tailored to address Decision-Making DM problems utilizing α,β,γ− neutrosophic information. This innovative structure incorporates three essential parameters, namely α, β, and γ, which play an essential role in the assessment of alternatives, adhering to the condition $\psi^{\alpha }+\eta^{\gamma }+\theta^{\alpha }≼3$, where α and β≽1 and γ≽1 is Least Common Multiple of α and β. The primary objective of this framework is to overcome the limitations associated with indeterminacy degrees, offering decision-makers a more flexible domain to simplify the handling of MCDM problems. The article introduces a new version of Neutrosophic Sets (NSs) and their basic operations, exploring aggregation operators such as α,β,γ− NWA, α,β,γ− NOWA, α,β,γ− NWG, and α,β,γ− NOWG operators to aggregate α,β,γ− neutrosophic information. Based on these operators, a detailed mathematical model is presented using step-by-step algorithms, and an illustrative example is constructed for the selection of the best location for a software office, showcasing the flexibility and reliability of the proposed model. Finally, through a comparative analysis with existing theories, we substantiate the authenticity and effectiveness of our proposed model.

Future research in α,β,γ− neutrosophic sets should focus on developing advanced aggregation methods [43], integrating with other mathematical frameworks, validating through empirical studies across domains [44], exploring new applications in AI and fostering interdisciplinary collaboration [45].

## Data availability

The accompanying manuscript does not contain any associated data. The paper only presents the written text and does not have any additional data that supports the claims and conclusions presented in the manuscript.

## CRediT authorship contribution statement

**Sumbal Ali:** Resources, Methodology, Formal analysis, Data curation. **Muhammad Rahim:** Writing – original draft, Methodology, Funding acquisition, Data curation, Conceptualization. **Sanaa A. Bajri:** Software, Project administration, Funding acquisition. **Sadique Ahmad:** Resources, Formal analysis, Data curation. **Rabab Alharbi:** Visualization, Validation, Supervision, Methodology. **Hamiden Abd El-Wahed Khalifa:** Methodology, Investigation, Formal analysis, Conceptualization.

## Declaration of generative AI and AI-assisted technologies in the writing process

During the preparation of this work the authors used CHATGPT in order to remove grammatical errors. After using this tool, the authors reviewed and edited the content as needed and takes full responsibility for the content of the publication.

## Declaration of competing interest

The authors declare that they have no known competing financial interests or personal relationships that could have appeared to influence the work reported in this paper.
